# Complete genome of *Rhodovulum* sp. HP10 isolated from the gastrointestinal tract of farmed shrimp

**DOI:** 10.1128/mra.00197-25

**Published:** 2025-05-20

**Authors:** Ha Thu Tran, Duong Huy Nguyen, Trang Thanh Pham, Tuyen Do Thi, Hang Dinh Thi Thu, Huyen Nguyen Thi Thu, Chintalapati Sasikala, Ha Hoang Chu, Yen Thi Hoang

**Affiliations:** 1 Institute of Biotechnology (IBT), Vietnam Academy of Science and Technology (VAST), Hanoi, Vietnam; 2 Graduate University of Science and Technology (GUST), VAST, Hanoi, Vietnam; 3 Thai Nguyen University of Sciences (TNUS), Thai Nguyen, Vietnam; 4 Smart Microbiological services, Hyderabad, India; University of Guelph, Guelph, Canada

**Keywords:** environmental microbiology, microbial genomics

## Abstract

The complete genome sequence of *Rhodovulum* strain HP10, which was isolated from the gastrointestinal tract of whiteleg shrimp, is presented here. The bacterium has one chromosome (4.17 Mb), one plasmid (62.43 kb), 4,128 coding sequences (CDSs), and 58 RNA genes and represents a novel species.

## ANNOUNCEMENT

*Rhodovulum* is a genus of purple non-sulfur anoxygenic phototrophic bacterium belonging to the class Alphaproteobacteria. Previous studies have shown that several genera of purple non-sulfur bacteria are associated with the removal of chemical oxygen demand (COD), nitrogen compounds, and H_2_S (1–3). These bacteria also possess antibacterial properties, particularly the ability to inhibit pathogenic bacteria in shrimp, including *Vibrio harveyi, Vibrio vulnificus,* and *Vibrio parahaemolyticus* (4). Furthermore, *Rhodovulum* species are used as probiotics in shrimp aquaculture, contributing to the improvement of pond environment and shrimp health (5, 6).

Here, we announce the genome sequence of strain *Rhodovulum* sp. HP10. The strain was isolated from the gastrointestinal tract of whiteleg shrimp, collected from a shrimp pond in Hai Phong City, Vietnam. The genome sequencing of *the* strain *Rhodovulum* sp. HP10 was performed using single-molecule, real-time (SMRT) DNA sequencing technology on the PacBio platform. The quality control reads and *de novo* assembly were performed using FastQC (http://www.bioinformatics.babraham.ac.uk/projects/fastqc/) and the long-read *de novo* assembler Flye version 2.9.5 (7). The contamination level of the assembled genome was assessed using the CheckM tool, which is integrated into the DDBJ Fast Annotation and Submission Tool web server (https://dfast.ddbj.nig.ac.jp/) (8). The quality and completeness of the assembled genome were evaluated using BUSCO version 5.8.0 (9). Annotation and gene prediction from the assembled genome were performed using the Rapid Annotations using Subsystems Technology (RAST) (10).

The complete genome consists of two contigs with a total length of 4,166,722 bp and a GC content of 67.19%. It has an N50 value of 4,104,297, an L50 of 1, and the largest contig length is 4,104,297 bp. The larger contig was identified as a chromosome, whereas the smaller contig was a plasmid with 62,425 bp in length. Genomic annotation reveals 4,186 genes, including 4,128 coding sequences (CDS) and 58 RNA genes. CheckM analysis indicates that the assembled genome has a completeness level of 99.3% and an estimated contamination of less than 1%.

To identify the taxonomy of strain *Rhodovulum* sp. HP10 and observe the relationships of the different species within the genus *Rhodovulum,* we conducted whole-genome-based taxonomic analyses with closely related type taxa using the Type Strain Genome Server (TYGS) (https://tygs.dsmz.de/) (11). Comparative analysis of digital DNA-DNA hybridization values between type strains of various *Rhodovulum* species and strain HP10 indicates that, with values below the 70% threshold, *Rhodovulum* sp. HP10 is a distinct species of the *Rhodovulum* genus.

## Data Availability

The genome sequencing data is deposited in the National Center for Biotechnology Information (NCBI) GenBank database under the Bioproject PRJNA1199221, the Biosample SAMN45869993, and the genome accession number CP180201 and CP180202. Additionally, the raw data is identified with the SRA number SRR32194844.
